# Adenoid hypertrophy detection inventory in children for primary care physicians and pediatricians

**DOI:** 10.1007/s00405-025-09350-8

**Published:** 2025-04-02

**Authors:** Fatih Kurt, Abdullah Belada, Buşra Oz, Sengul Cangur, Abdulkadir Kaya

**Affiliations:** 1 Pediatrics Clinic, Duzce University, Duzce, Turkey; 2 Ear, Nose and Throat Clinic, Duzce University, Duzce, Turkey; 3 Childand and Adolescent Clinic, Duzce University, Duzce, Turkey; 4 Biostatistics Department, Duzce University, Duzce, Turkey; 5 Family Medicine Clinic, Duzce University, Duzce, Turkey; 6 Department of Pediatrics, Duzce University, Duzce, 81000 Turkey

**Keywords:** Adenoid hypertrophy, Inventory, Nasopharyngoscopy, Snoring

## Abstract

**Objective:**

Adenoid tissue consists of clusters of lymphoid tissue within the nasopharynx and can cause symptoms due to obstruction when hypertrophied. The gold standard for diagnosis is endoscopic nasopharyngoscopy, but it is not always readily available. This study aims to develop an inventory that primary care physicians and pediatricians can use to predict the degree of adenoid hypertrophy clinically, facilitating the planning of patient follow-up and treatment.

**Study design:**

A diagnostic test study.

**Settings:**

tertiary referral hospital.

**Methods:**

The study involved 123 cases, with 82 in the patient group and 41 in the control group. Evaluation encompassed demographic characteristics, history, and physical examination findings. Additionally, a child psychiatrist assessed cases neurocognitively, behaviorally, and psychologically. Finally, cases underwent endoscopic nasopharyngoscopy by an ENT specialist, recording adenoid sizes and choanae narrowing. Multinomial Logistic Regression (MLR) analysis determined the most suitable model for the clinical inventory.

**Results:**

Snoring, restless sleep, noisy breathing, recurrent throat infections, and recurrent rhinosinusitis constitute the items of the clinical inventory. The average score of relevant items categorized patients into absent and mild, moderate, and severe groups. The area under the ROC curve for average scores of the inventory was 0.67, significantly surpassing the probability of random assignment (0.17). The inventory’s accuracy rate was 70%.

**Conclusion:**

This user-friendly and highly accurate inventory aids in predicting obstruction degree in patients. Primary care physicians and pediatricians can effectively manage follow-up and treatment, referring cases requiring surgery to an ENT specialist based on the inventory results.

## Introduction

Adenoid tissues, located at the junction of the roof and posterior wall of the nasopharynx, are lymphoid tissue clusters that are included in the Waldeyer ring and constitute an important part of the child’s local and systemic immune mechanism [1]. The surface is covered with pseudostratified ciliated columnar epithelium. Physiologically, they exhibit a growth pattern by initially doubling in size compared to the adult dimensions during childhood. They reach their maximum size by the age of 6, begin to decrease in size at the age of 10, and disappear during adolescence. However, pathological hypertrophy may occur due to bacterial or viral infections, abnormal immune reactions, environmental exposures, hormonal factors, genetic factors or allergy-related factors [2]. The prevalence of adenoid hypertrophy in the general pediatric population is approximately 34% [3]. The adenoid-palatal space narrows depending on the degree of adenoid hypertrophy. As a result of this narrowing, conditions such as sleep apnea, nasal cavity obstruction, mouth breathing, adenoid facies, malocclusions, changes in voice tone, snoring, exudative middle ear infection due to eustachian tube blockage and hearing loss may develop [4]. Additionally, it has been reported to cause impaired neurocognitive performance and behavioral and psychological problems [5].

Medical history and physical examination are crucial in the diagnosis of adenoid hypertrophy. Although endoscopic nasopharyngoscopy is the gold standard method for diagnosis, ultrasound and lateral radiographs of the nasopharynx can be used in some patients [4]. It is not always possible to reach an ear, nose and throat (ENT) specialist for nasopharyngoscopic evaluation. Early treatment of adenoid hypertrophy prevents sleep apnea and recurrent infections, reduces neurodevelopmental problems, and improves quality of life. Therefore, there is a need for an inventory predicting the degree of adenoid hypertrophy in children. This approach would enable early treatment and prevention of complications. In this study, our aim was to develop an inventory that can clinically predict the degree of adenoid hypertrophy, facilitating the planning of follow-up and treatment by primary care physicians or pediatricians and identifying patients who need to be referred to ENT clinics.

## Materials and methods

A total of 123 patients were enrolled in the study, 82 patients were in the study group, and 41 were in the control group. The demographic characteristics of these patients (age, sex, etc.), medical history and physical examination findings, such as adenoid face, mouth breathing during sleep, snoring, sleep apnea, daytime mouth breathing, hyponasal speech, recurrent otitis media and family history of adenoid hypertrophy, were evaluated. A comprehensive psychiatric examination was conducted for all participants by a child and adolescent psychiatry specialist. Since a separate scale was required for the evaluation of each psychiatric symptom used in the survey, the symptoms were assessed based on information obtained from the family and the child, as well as the results of the psychiatric examination. Participants’ aggressive behaviors in school, family, and external environments were evaluated as ‘none,’ ‘mild,’ ‘moderate,’ and ‘severe.’ Similarly, anxiety symptoms, daytime sleepiness, attention deficit (inattention), happiness, and school performance (report card average: >85 = high, 60 < middle < 85, < 60 = low) were evaluated based on the psychiatric examination and the information obtained from the family and the child. Finally, the ENT specialist performed endoscopic nasopharyngoscopy on the patients. Adenoid sizes and the degree of choanal narrowing were recorded for each patient. The patients were classified as grade 3 (50%-75%) for moderate obstructions or grade 4 (75%-100%) for severe obstructions. The control group consisted of asymptomatic patients with grade 1 (0%-25%) and grade 2 (25%-50%) obstructions. Patients with septal deviation identified during endoscopic examination, patients with acute rhinosinusitis, patients with uncontrolled allergic rhinitis or patients with palatine tonsils greater than grade 2 were excluded from the study.

Written informed consent was obtained from the mothers and relatives of the patients.

This research involving human subjects complied with all relevant national regulations and institutional policies and was conducted in accordance with the tenets of the Helsinki Declaration. This study was approved by Duzce University Faculty of Medicine Ethics Committee (Decision no:2022/189, Approval Date:07.11.2022).

### Statistical analysis

Quantitative variables are presented as the mean ± standard deviation, while qualitative data are presented as counts and percentages. Normality assumptions for quantitative variables were examined using skewness, kurtosis coefficients and the Shapiro‒Wilk test. The assumption of homogeneity of group variances was checked using the Levene test. One-way ANOVA was used for intergroup comparisons of quantitative variables. The relationship between categorical variables was examined using the Pearson chi-square test. The most suitable model was established through multinomial logistic regression (MLR) analysis of the Adenoid Hypertrophy Detection Inventory in Children (AHDIC). Three-way and two-way receiver operating characteristic (ROC) curve analyses were applied to determine appropriate threshold values for the mean AHDIC score when adenoid hypertrophy was classified as absent and mild, moderate, or severe. Pearson correlation analysis was employed to investigate the criterion validity of the AHDIC. All the statistical analyses were conducted using SPSS 22 software and the R CRAN 2023.06.0 programming language, and *p* < 0.05 was considered to indicate statistical significance.

## Results

Adenoid hypertrophy of 123 cases was measured by endoscopic examination. Forty-two of the cases (34.1%) were in the absent and mild group, 41 (33.3%) were in the moderate group, and 40 (32.5%) were in the severe group. Among the children, 58 (47.2%) were girls and 65 (52.8%) were boys, and the mean age was 7.15 ± 2.21 years. There was no significant difference between the groups in terms of age or sex (*p* > 0.05; Table 1). The detailed characteristics of the children were provided in Table 1.

**Table 1:** Distribution of children participating in the study according to the degree of adenoid hypertrophy

		Group				*p*
		Absent and Mild (Group1)	Moderate (Group 2)	Severe (Group 3)	Total	
Age (year)		7.33 ± 2.13	7.51 ± 2.13	6.58 ± 2.30	7.15 ± 2.21	0.128
Gender	Girl n (%)	23 (54.8%)	22 (53.7%)	13 (32.5%)	58 (47.2%)	0.077
	Boy n (%)	19 (45.2%)	19 (46.3%)	27 (67.5%)	65 (52.8%)	
Obstruction Degree (%)		29.52 ± 17.24	64.88 ± 5.06	86.25 ± 7.05	59.76 ± 26.03	-

To determine AHDIC, the following criteria were used: adenoid face (absent, present), dental malocclusions (absent, present), sleeping with open mouth (absent, < 50% of sleep time, > 50% of sleep time), snoring (absent, mild (< 25%), moderate (25-50%), severe (> 50%), restless sleep (absent, present), sleep apnea (absent, present), difficulty waking up in the morning (absent, present), and daytime mouth breathing (absent, present). ), noisy breathing (absent, present), aggression (absent, mild, moderate, severe), anxiety (absent, mild, moderate, severe), daytime sleepiness (absent, present), inattention (absent, mild, moderate, severe), decrease in joyfulness (absent, mild, moderate, severe), school success (high, moderate, low), developmental delay (absent, present), hyponasal speech (absent, present), chronic otitis media with effusion (absent, yes), recurrent acute otitis media (absent, yes), recurrent lower respiratory tract infections (absent, yes), allergic rhinitis (absent, yes), history of asthma (absent, yes), history of atopy (absent, present), recurrent sore throat infection (absent, present), chronic nasal congestion (absent, present), chronic rhinosinusitis (absent, present), recurrent rhinosinusitis (absent, present), halitosis (absent, mild, moderate, strong), paroxysmal sneezing (absent, present), hearing loss (absent, present), dysphonia (absent, present), attention deficit (absent, mild, moderate, severe), hyperactivity (absent, mild, moderate, severe), cognitive capacity (normal, borderline, intellectual disability), learning disability (absent, mild, moderate, severe), forgetfulness (absent, mild, moderate, severe), laryngeal reflux (absent, present), difficulty falling asleep (absent, present), enuresis nocturna (absent, present), adenoid hypertrophy in the family (absent, present), total of 40 clinical signs or symptoms were evaluated.

A significant multinomial logistic regression model (χ²=114.15, *p* < 0.001; Nagelkerke R²=0.68; accuracy ratio = 0.70) was generated through a series of models to identify the clinical signs or symptoms that best distinguished between the three groups (absent and mild, moderate, severe) classified by adenoid hypertrophy size. According to this model, clinical signs or symptoms such as snoring (absent = 0 points, mild (< 25%) = 1 point, moderate (25–50%) = 2 points, severe (> 50) = 3 points), restless sleep (absent = 0 points, present = 1 point), noisy breathing (absent = 0, present = 1), recurrent throat infections (absent = 0, present = 1), and recurrent rhinosinusitis (absent = 0, present = 1) constitute the items of this inventory. The inventory score is obtained by calculating the average score of the relevant items. AHDIC is presented in Table 2.

**Table 2:** Adenoid hypertrophy determination inventory in children

Signs and Symptoms	0 point	1 point	2 point	3 point
1. Snoring (per hour of sleep) (6)	Absent	Mild (< 25%)	Moderate (25-50%)	Severe (> 50%)
2. Restless sleep	Absent	Present		
3. Noisy breathing (7)	Absent	Present		
4. Recurrent throat infections	Absent	Present		
5. Recurrent rhinosinusitis	Absent	Present		
**Average Score:……………………** Absent and Mild (Average Score < 0.3) Moderate (0.3 ≤ Average Score < 0.9) Severe (Average Score ≥ 0.9)				

Restless sleep: Frequent changes in position or motor movements involving large muscle groups during sleep [8]

Recurrent throat infections: More than 7 episodes per year in the last 1 year, or more than 5 episodes per year in the last 2 years of upper respiratory tract infections [9]. Recurrent rhinosinusitis: Four or more episodes of acute rhinosinusitis lasting at least ten days each per year, without persistent symptoms between these different attacks [10]

## Adenoid hypertrophy detection inventory in children (AHDIC) performance

**Fig. 1:**
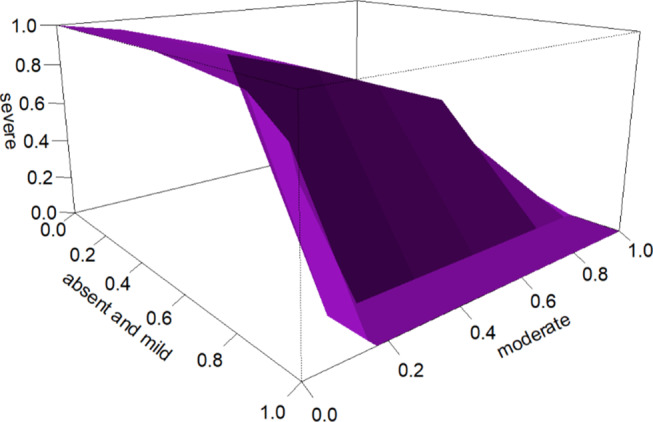
3D ROC curve plot for the average score of the Adenoid Hypertrophy Detection Inventory in Children

The performance results of the childhood adenoid hypertrophy detection inventory when patients were classified according to the gold standard as absent and mild, moderate, or severe are presented in Table 3. In situations where these three classes existed, the volume under the ROC curve for the average AHDIC score was 0.67 (standard error (SE) = 0.052, test statistics = 9.64, *p* < 0.001). This obtained value is significantly high and meaningful, exceeding 0.17 (probability of an individual being randomly assigned to the correct class). The accuracy of the AHDIC diagnostic test for classifying patients into absent and mild, moderate and severe groups was 70%. According to this accuracy, the cutoff value that best distinguishes the mild and moderate groups was determined to be an average inventory score of 0.3, while the best cutoff value that distinguishes the moderate and severe groups was found to be an average inventory score of 0.9. Figure 1 shows the 3D ROC curve plot for the average AHDIC score. Binary comparisons (post hoc) were conducted to examine the discriminative ability of the AHDIC between each pair of AH classes. Accordingly, when evaluating the ability of the AHDIC to distinguish between mild and moderate cases, the area under the curve (AUC) was found to be 0.93 (SE = 0.027, *p* < 0.001; threshold = 0.3, sensitivity = 0.83, specificity = 0.86 for an average score). The evaluation of the success of the AHDIC in distinguishing between the mild and severe groups yielded an AUC of 0.97 (SE = 0.015, *p* < 0.001; threshold = 0.3; sensitivity = 0.95; specificity = 0.86 for an average score). When assessing the success of the AHDIC in distinguishing between the moderate and severe groups, an AUC of 0.74 (SE = 0.055, *p* < 0.001; threshold = 0.9; sensitivity = 0.55; specificity = 0.85 for an average score) was obtained.

**Table 3:** Performance results of the adenoid hypertrophy detection inventory in children in three grades (absent and mild, moderate, severe)

	AHDIC Average point
VUS	0.67
Standard error of VUS	0.052
Variance of VUS	0.003
Test statistics	9.64
p value	< 0.001
95% Logit Confidence Interval	0.56–0.76
First coordinate value	0.86
Second coordinate value	0.68
Third coordinate value	0.55
Accuracy	0.70
The best first threshold value (c1)	0.3
The second first threshold value (c2)	0.9

AHDIC: Adenoid Hypertrophy Detection Inventory in Children

VUS: Volume under ROC surface

In all the children, there was a statistically significant positive correlation between AHDIC and adenoid hypertrophy size (*r* = 0.73, *p* < 0.001). In the moderate + severe group, a positive and significant correlation was found between AHDIC and adenoid hypertrophy size (*r* = 0.45, *p* = 0.003), while in the absent and mild group, no significant correlation was observed (*p* > 0.05). According to these results, the criterion validity of the AHDIC was established.

## Discussion

Adenoids are a group of lymphoid tissue located in the upper part of the nasopharynx at the base of the sphenoid bone. They typically reach their largest size around the age of 6 and then often regress, primarily disappearing during adolescence [4]. Adenoid hypertrophy is a clinical condition in which adenoids grow to pathological sizes, causing obstruction and significantly impacting the quality of life of children. It can lead to obstructive sleep apnea syndrome, chronic sinusitis, effusion-based otitis media, speech and articulation disorders, and disruptions in physical and intellectual development [2].

Sizer et al. reported that children who underwent adenoidectomy experienced significant decreases in scores on the Children’s Anxiety Screening Scale and Children’s Sleep Scale, along with a notable increase in scores on the Children’s Quality of Life Scale after the operation. These findings suggest that treating adenoid hypertrophy can potentially reduce neurobehavioral problems and enhance quality of life by preventing sleep apnea and recurrent infections [5].

Therefore, developing an inventory that predicts the degree of adenoid hypertrophy in children is crucial. This approach not only aids in the early treatment and prevention of complications for patients who cannot access an ENT specialist but also helps prevent unnecessary referrals from patients whom primary care physicians and pediatricians can manage to ENT clinics. This is of utmost importance in the efficient utilization of healthcare resources.

Snoring is the harsh sound produced during airflow due to collapsible soft tissues in the nasal and pharyngeal regions. Snoring occurring for a minimum of three nights per week is referred to as habitual snoring and is indicative of sleep-disordered breathing. It has been reported that 77% of children with adenoid hypertrophy complain of snoring, and 69% of children with snoring exhibit adenoid hypertrophy [11, 12]. In our study, mild snoring was present in 3% of patients in Group 1. In Group 2, 39% of the patients had mild snoring, 12% had moderate snoring, and 14% had severe snoring. Among patients in Group 3, 20% had mild snoring, 12% had moderate snoring, and 47% had severe snoring complaints.

According to the 2014 definition provided by the American Academy of Sleep Medicine, restless legs syndrome (RLS) is a clinical condition characterized by the occurrence of more than 5 significant body movements per hour, lasting for at least 3 months and occurring at least 3 times a week. Parents often report that their children exhibit constant body movements, disrupted bedclothes, or falling out of bed during sleep [13]. Numerous studies have demonstrated an association between adenoid hypertrophy and restless legs syndrome. In a study conducted by Inönü-Sakallı et al., Restless Legs syndrome was reported to be present in 75% of patients with adenoid hypertrophy, whereas it was found in 7.5% of patients in the control group [14]. In our study, RLS was observed in 26% of patients in Group 1, 70% of patients in Group 2, and 90% of patients in Group 3.

Noisy breathing is typically identified as a symptom during the first year of life. The most common cause of concern for both parents and clinicians in this situation is laryngomalacia. In older children, adenoid hypertrophy plays a significant role. Approximately 60% of patients with adenoid hypertrophy complain of loud breathing [15]. In our study, none of the patients in Group 1 exhibited a complaint of loud breathing. In Group 2, this complaint was observed in 29% of patients, and in Group 3, it was present in 42% of patients.

Tonsillopharyngitis is one of the most common reasons for clinical visits. According to Paradise, recurrent tonsillopharyngitis is defined as a minimum of 7 attacks per year, at least 5 attacks per year for two consecutive years, or at least 3 attacks per year for three consecutive years. It is generally caused by viral infections [16]. Adenoid and tonsil hypertrophy have been identified as significant factors in recurrent tonsillopharyngitis, and patients who are severely affected by these conditions benefit from tonsillectomy and adenoidectomy [17]. In our study, a history of recurrent tonsillopharyngitis was present in 21% of patients in Group 1, 63% of patients in Group 2, and 65% of patients in Group 3.

The association between recurrent acute sinusitis and adenoid hypertrophy, another parameter in our inventory, has been frequently reported. Recurrent acute rhinosinusitis is defined as the occurrence of rhinosinusitis attacks, each lasting for at least 10 days with symptom-free intervals, at least 4 times per year [10]. Approximately 57% of patients with adenoid hypertrophy experience recurrent sinusitis [18]. In our study, a history of recurrent acute rhinosinusitis was present in 9% of patients in Group 1, 31% of patients in Group 2, and 57% of patients in Group 3.

The associations of chronic nasal congestion, daytime mouth breathing, recurrent otitis media, and sleep apnea with adenoid hypertrophy have been reported in many studies [1]. However, these parameters were excluded due to their inadequate performance in distinguishing between the three groups in our study.

There have been a limited number of studies predicting the degree of adenoid hypertrophy through clinical evaluation. In a study involving 90 patients, parameters such as difficulty breathing while asleep, snoring, and apnea were scored. Subsequently, the adenoid sizes of the patients were evaluated first via lateral nasopharyngeal X-ray and subsequently via nasopharyngeal endoscopy. One study reported a significant correlation between clinical score and the degree of endoscopic hypertrophy [19].

In the scoring system reported by Bitar et al., the parameters included mouth breathing, snoring, restless sleep, frequent awakening at night (at least 3 times per night due to respiratory discomfort or distress), and obstructive breathing during sleep [20].

In both of these studies, a set of parameters was initially identified, followed by an attempt to determine the degree of obstruction caused by adenoid hypertrophy through these symptoms. In our study, however, we compiled a list of 40 symptoms that have been previously reported to be associated with adenoid hypertrophy and questioned each of our patients about these symptoms. We aimed to create an optimal scale from these symptoms to determine adenoid size and then compare it with the gold standard.

Both of these studies have been utilized to distinguish between mild obstruction and moderate-severe obstruction. Two-way studies have not differentiated between moderate and severe obstruction. However, our study classified obstructions into three groups: mild, moderate, and severe. Distinguishing between moderate and severe obstructions is crucial for medical treatment. In a study by Ras et al., patients with adenoid hypertrophy and obstructions in the range of 50–75% responded better to medical treatment than did those with obstructions in the range of 75–100%, and a significant difference was observed between the two groups [21].

## Conclusion

Adenoid hypertrophy is one of the most common reasons why children seek ENT clinics. However, a significant portion of patients with mild to moderate obstruction can be managed and treated by primary care physicians and pediatricians. It is important to determine which patients can be monitored without medication, which patients will receive medical treatment, and which patients will be referred for surgery. With the development of this easy-to-apply and highly accurate inventory, patients were categorized into three groups based on the degree of obstruction. Primary care physicians and pediatricians were enabled to follow up and treat patients who could not reach an ENT specialist.
